# T-cell Metabolism as a Target to Control Autoreactive T Cells in β-Cell Autoimmunity

**DOI:** 10.1007/s11892-017-0848-5

**Published:** 2017-03-16

**Authors:** Carlotta Bordignon, Adriana Canu, Aleksandra Dyczko, Serena Leone, Paolo Monti

**Affiliations:** 1 San Raffaele Diabetes Research Institute, IRCCS San Raffaele Scientific Institute, 20131 Milan, Italy; 2 San Raffaele Vita-Salute University, Via Olgettina 58, 20131 Milan, Italy

**Keywords:** Type 1 diabetes, T cells, Immune-metabolism, Autoimmunity

## Abstract

**Purpose of review:**

An increasing body of evidence indicates that bio-energetic metabolism of activated T cells is a potential target to control the autoimmune response in type 1 diabetes (T1D).

**Recent findings:**

T-cell activation and proliferation is linked to the cell capacity to provide sufficient energy and biosynthesis molecules to support T-cell growth and division. This makes T cells susceptible to metabolic inhibition for the control of the T-cell response. There is a wide therapeutic arsenal of metabolic inhibitors, including novel classes of drugs that have become recently available.

**Summary:**

With the current knowledge and availability of metabolic inhibitors, we are now in the position to design a metabolic inhibition strategy to determine whether targeting of autoreactive T cells is an effective strategy to control the process of β-cell destruction in T1D.

## Introduction

Autoreactive T cells have a pivotal pathogenic role in the autoimmune process of β-cell destruction. Consequently, autoreactive T cells are an ideal target for immunotherapy strategies aiming to prevent or delay the onset of the disease. In patients with type 1 diabetes (T1D), autoreactive T cells are largely refractory to standard immunosuppressive [1] and immunomodulatory strategies [2–4], and the identification and characterization of novel target pathways to control the T-cell response to β cells is still a strong need. In recent years, there was an ever-growing appreciation of how every aspect of the T-cell response is tightly linked to bio-energetic metabolism [5]. Resting T cells need basal energy production and biosynthesis to support house-keeping functions and homeostasis. On the other hand, activation, clonal expansion, and acquisition of effector functions of T cells are energetic and biosynthetic demanding processes. Immediately upon activation, T cells strongly upregulate the anabolic process of glycolysis even in the presence of oxygen (Warburg effect). Later, also oxidative phosphorylation fueled by amino acids is upregulated to sustain proliferation. The extraordinary energetic and biosynthetic needs during expansion also make T cells vulnerable to apoptosis and unresponsive if these needs are not fulfilled. This opened a new perspective on the potential to control the T-cell response by manipulating metabolism with drugs [6]. A consistent number of publications indicate that metabolic inhibition is an effective strategy to control the T-cell response in preclinical models of autoimmunity and transplantation. In this paper, we will review recent literature on T-cell metabolism to understand whether we can envisage a translation of this novel approach to control β-cell autoimmunity. An ideal immunotherapy for T1D has to be nontoxic, limited in time, selective on autoreactive T cells, and provide long-term effect in the control of β-cell autoimmunity. We will attempt to design a potential strategy of metabolic inhibition that satisfy these needs, considering the current knowledge on T-cell metabolism and the current availability of drugs targeting metabolism.

## Brief Overview of Metabolic Pathways

Cells regulate the activity of metabolism to couple generation of energy and biosynthesis intermediates for survival and proliferation [7]. Glycolysis and oxidative phosphorylation (OXPHOS) are the two processes by which cells produce chemical energy in the form of ATP. Glucose is one of the substrates from which cells produce energy, and glycolysis begins with the uptake of extracellular glucose. Glycolysis converts glucose into pyruvate via a series of intermediate metabolites that can enter the pentose phosphate pathway, thus contributing to biosynthesis for cell growth. In the mitochondria, pyruvate can be converted into acetyl-CoA, which enters the tricarboxylic acid (TCA) cycle. Alternatively, pyruvate can be converted in the cytoplasm into lactate, which is excreted from the cell. In addition to energy and biosynthesis intermediates, glycolysis also allows the reduction of NAD+ to NADH, thus contributing to the cell redox balance. Lipids enter the fatty acid oxidation (FAO) process providing intermediates that enter the TCA cycle. Also, amino acids such as glutamine can be metabolized to produce intermediates for the TCA cycle. Glutamine is converted into glutamate by glutaminase. Glutamate enters the mitochondria through the mitochondrial glutamate transporter and is used in the TCA cycle. Oxidation of substrates in the TCA cycle produces the coenzymes NADH and FADH2 and gives electrons to the electron-transport chain to sustain OXPHOS. The TCA cycle also produces intermediates for biosynthetic pathways, such as citrate, which is used for fatty acids synthesis. ATP production by aerobic glycolysis is 10 times less efficient than by OXPHOS. The T-cell decision to use glycolysis or OXPHOS is dictated by the state of activation to find the optimal balance for ATP production, biosynthetic substrates, and redox condition.

## Metabolism of Quiescent and Activated T Cells

Quiescent, non-proliferating T cells have low energetic and biosynthetic demand and use glucose, fatty acids, and amino acids to fuel ATP generation by the catabolic process of OXPHOS. Survival of naïve T cells depends on interleukin-7 signaling [8], which acts as a master regulator of metabolism by promoting extracellular glucose uptake via GLUT-1, and by transcriptional control of the glycolytic enzyme exokinase II gene. In the absence of IL-7, naïve T cells reduce metabolic activity and rapidly undergo atrophy. Resting memory T cells have a greater mitochondrial mass and an increased spared respiratory capacity (SRC) than that of naïve T cells [9]. This is closely related to the capacity of memory T cells to provide immediate metabolic activation for rapid recall response. Memory T cells synthetize lipids that serve to fuel FAO in the mitochondria starting either from extracellular glucose or glycerol. Glycerol is acquired from the extracellular environment through the IL-7-dependent expression of the glycerol channel aquaporin 9 (AQP9) [10••]. Glycerol is subsequently used for fatty acid esterification and triglyceride (TAG) synthesis and storage, which appear to be a critical process for long-term memory T-cell survival.

**Figure 1:**
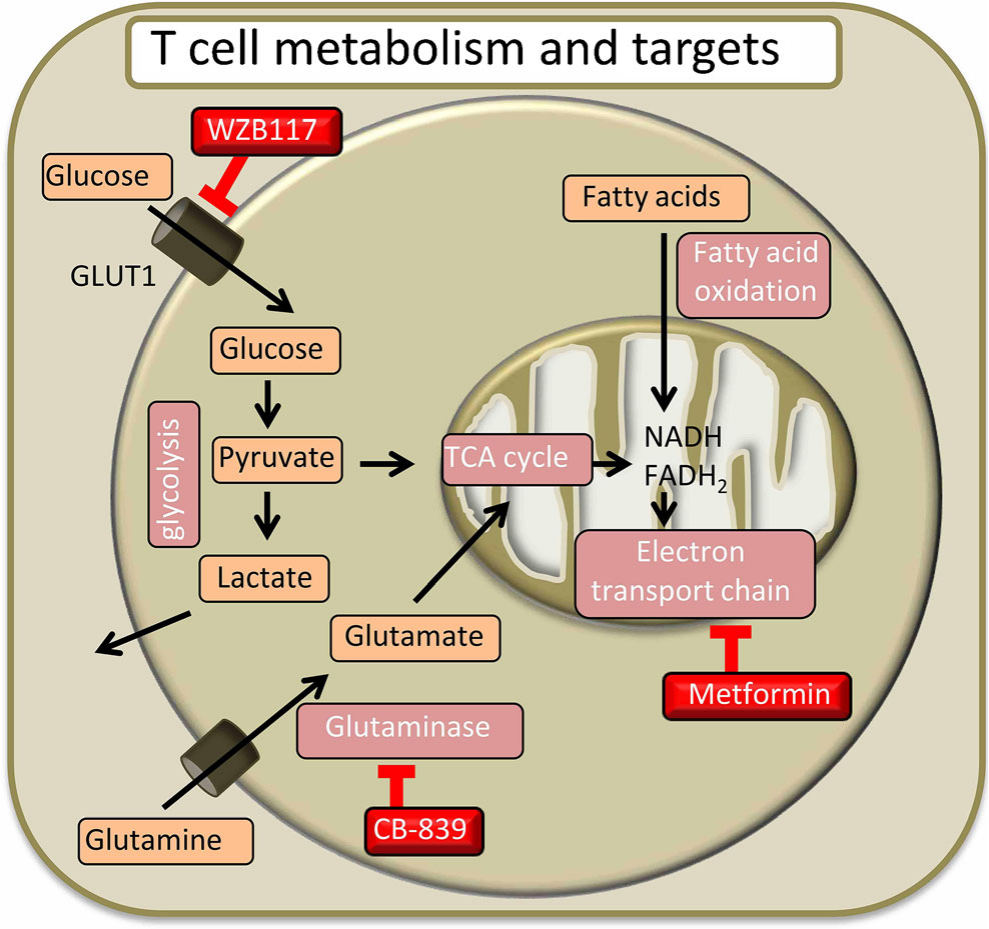
T-cell metabolism and inhibitors. Upon antigen recognition, activated T cells activate glycolysis and subsequently mitochondrial oxidative metabolism to produce energy and biosynthesis intermediates for cell growth and proliferation. The GLUT1 inhibitor WZB117, the ETC inhibitor metformin, and the glutaminase inhibitor CB839 are potential candidate molecules to induce a metabolic blockade. *GLUT1* glucose transporter 1, *TCA* tricarboxylic acids

Upon antigen recognition, signals from the TCR, with proper co-stimulation and cytokine signals, lead to T-cell proliferation and activation. The rate of cell growth and division of activated T cells during clonal expansion is unique in the body and requires an extraordinary amount of energy and substrates to build the “blocks” for growth. One of the most striking changes to affect T cells upon antigenic stimulation is the metabolic reprogramming (Fig. 1). T cells significantly enhance glucose uptake, through increased expression of GLUT1 [11]. A parallel production of lactate indicates that pyruvate has been reduced rather than undergooxidation in the TCA cycle. The initial burst of glycolysis appears to be necessary for the production of biosynthetic precursor molecules. Catabolic OXPHOS is actively suppressed as the production of ATP from mitochondria would increase the ATP:ADP ratio reducing the glycolytic rate. The metabolism of T cells early after antigen encounter largely relies on glycolysis even in the presence of oxygen to support mitochondrial oxidations. This is reminiscent of the Warburg effect, which is a hallmark of cancer cell metabolism and was extensively studied as a target to affect cell growth and replication. After the acute phase of activation characterized by glycolysis, oxidative mitochondrial processes increase in chronically activated T cells [10••]. Pyruvate is oxidized in the TCA cycle and pyruvate dehydrogenase is down-regulated. In addition to pyruvate, critical substrates used by activated T cells are glutamine and fatty acids [12•]. With respect to the signaling pathways involved in metabolic reprogramming, TCR activation along with CD28 co-stimulation triggers the activity of phosphatidylinositol 3′-kinase (PI3K) and downstream activation of the serine-threonine kinase Akt. Akt promotes the localization of GLUT1 to the plasma membrane to facilitate glucose uptake, and increases the activity of the glycolytic enzymes hexokinase and phosphofructokinase [13]. In addition, Akt promotes the mammalian target of rapamycin (mTOR) activity by relieving inhibition of RhebGTP by the TSC1-TSC2 complex [14]. mTOR activation modulates the rate of protein synthesis by regulating the process of cap-dependent translation and by increasing the surface expression of amino acid transporters [15]. The mTOR pathway leads to the expression of downstream transcriptional regulators, including c-Myc, and the hypoxia inducible factor 1α, (HIF-1α). Activation of c-Myc is associated with the expression of enzymes that promote aerobic glycolysis, and upregulation of glutaminolysis to enhance production of amino acids, lipids, and nucleic acids necessary for cell growth [16]. HIF-1α is a transcription factor that responds to oxygen levels, acting as an important promoter of glucose uptake and glycolysis in many cell types, including T cells [17].

Although much of the attention on metabolic changes in T cells upon activation has focused on the early upregulation of glycolysis, recent findings have revealed the role of oxidative metabolism in the mitochondria. Mitochondrial OXPHOS is activated later than glycolysis and is active in chronically activated T cells, such as alloreactive T cells mediating graft vs host disease [18]. It was proposed that after the initial phase of activation, T cells undergo metabolic stress, perhaps due to limitation in nutrients, cytokines, and antigenic stimulation switching from glycolysis to other catabolic processes, like fatty acid oxidation [19]. This switch is a key step to the survival of proliferating clones to develop long-lived memory cells during the contraction phase of the T-cell response. In addition to ATP production, mitochondrial oxidation can influence the T-cell proliferation rate though production of reactive oxygen species (ROS) [20•]. The lymphocyte expansion molecule (LEM) controls the levels of OXPHOS complexes and regulates the production of pro-proliferative ROS in the mitochondria of activated T cells.

## Controlling the T-Cell Response Through Metabolism

The rapid and extensive clonal expansion capacity of T cells is unique among cells in the body. The extraordinary energetic and biosynthetic effort needed to support these processes makes proliferating T cells particularly amenable to metabolic manipulation. Metabolic manipulation not only influences proliferation but also differentiation into different T-cell subsets, the generation of memory T cells, and the capacity to respond to recall antigen in the long term. Several studies have shown that molecules interfering with T-cell metabolism influence the final outcome of the T-cell response. The mTOR inhibitor rapamycin, which is widely used as an anti-proliferative immunosuppressive drug, was shown to promote the generation of memory T cells in the CD8+ T-cell subset [21]. One possibility is that mTOR blocking induces a switch to catabolic metabolism in proliferating naïve T cells. The anti-diabetes drug metformin, which act as activator of AMPK and inhibitor of the electron transport chain in the mitochondria, also enhances CD8 T-cell memory generation [19]. It is likely that the effect of both rapamycin and metformin on memory T-cell generation is a consequence of a metabolic manipulation. Indeed, specific induction of FAO results in an increased generation of memory T cells and indicates that promoting catabolic pathways is crucial to the development of long-lived memory cells. A population of memory T cells with stem cell-like properties has been recently identified and characterized in mice, non-human primates, and humans, and named stem cell memory T cells [22]. The existence of autoreactive Tscm precursors has been hypothesized also in type 1 diabetes (T1D) [23]. Tscm can be generated by activation of naïve T cells in the presence of the glycogen synthetase kinase beta (GSK3b) inhibitor TWS119 [24]. In addition, Tscm can be generated by culturing naïve T cells in the presence of IL-7 and IL-15, which are modulators of T-cell glycolytic and mitochondrial metabolism [25]. The availability of nutrients also impacts T-cell activation and proliferation. Glucose is a fundamental nutrient for CD8+ T-cell cytolytic activity and cytokine production. Consequently, these functions are impaired in glucose-limiting conditions, such as in the tumor microenvironment. Low concentration of extracellular glucose is a hallmark of the milieu of tumors with high glycolytic rate. CD8+ T cells isolated from these tumors have impaired interferon-γ production and reduced mTOR activation compared with that of T cells from tumors with basal levels of glycolysis [26•]. T-cell survival and function also depend on availability of amino acids. In vitro T-cell proliferation and cytokine production is severely impaired by depletion of glutamine from the culture medium and it is likely that competition for glutamine can influence the T-cell response [27••]. The role of other amino acids is currently under investigation. Deficiency in the leucine transported Slc7a5 (LAT1) was shown to prevent proliferation and effector functions of both CD4+ and CD8+ T cells [28]. The mechanism involves a defective activation of mTOR and impairment of the uptake of glucose and glutamine to fuel metabolic processes.

Also by-products generated during metabolic processes can affect the T-cell response. Lactate is produced during glycolysis and then excreted in the extracellular milieu. Lactate promotes the differentiation of Th17 cells and inhibits the cytolytic activity of CD8+ T cells [29]. Indoleamine-2,3-dioxigenase mediates tryptophan catabolism and produces kynurenine, which induces the generation of regulatory T cells and acts as a potent suppressor of effector T-cell proliferation [30]. These data show how metabolic activity is involved in the T-cell fate during an immune-response, and open the possibility to control the T-cell response by targeting metabolic processes with specific drugs.

## Drugs and Molecules Targeting Metabolism

The possibility to target metabolic pathways in T cells is relatively recent; however, this therapeutic option has been extensively explored to treat cancer, and several drugs and molecules have been developed and become available (Table 1). Two important inhibitors of glycolysis are 2-deoxy-D-glucose (2DG) and 2-fluoro-deoxy-D-glucose (2FDG) [31]. The structure of 2DG is identical to that of D-glucose, except that the C-2 hydroxyl group is replaced with hydrogen. As for D-glucose, 2DG is taken up by cells through trough GLUTs transporters. In the cell, 2DG is phosphorylated by hexokinase (the first enzyme of the glycolytic pathway) to form 2DG-6-phosphate, which cannot be subsequently processed through the glycolytic pathway. 2DG-6-phosphate accumulates in the cell and competes with glucose-6-phosphate for phosphoglucose isomerase (PGI) in the subsequent reversible glycolytic step. Thus, the primary way by which 2DG blocks glycolysis is by competitive inhibition of PGI. 2FDG is more potent than 2DG in inhibiting glycolysis. This correlates with the closer structural similarity of 2FDG to glucose than 2DG. This increases the binding of 2FDG to the catalytic subunit of hexokinase and consequently an increased production of 2FDG-6-phosphate and enhanced inhibition of PGI. Both 2DG and 2FDG are highly toxic compounds and their mechanism of inhibition of the glycolytic enzymes causes inhibition of glucose metabolism virtually in every cell in the body. A novel class of small molecules that inhibit glycolysis by blocking glucose uptake through the GLUT1 transporter has recently been developed. The advantage of these compounds is that GLUT1 is used for glucose uptake predominantly by neurons, activated T cells, and certain types of cancer cells, and they should not interfere with the glycolytic pathway of other cells using different glucose transporters. STF-31 is a GLUT1 inhibitor, which showed a selective toxicity of renal cancer cells expressing GLUT1 and using glycolysis for ATP production [32]. Normal kidney cells are not strictly dependent on glycolysis, use GLUT2 for glucose uptake, and are therefore insensitive to STF-31 toxicity. When used in a mouse model, STF-31 efficiently blocked tumor growth without significant toxic effect on other organs. Similarly, WZB117 is a small molecule competitively inhibiting GLUT1-mediated glucose transport [33]. WZB117 was shown to kill lung and breast cancer cells without affecting normal cells. As for STF-31, in vivo treatment of animal models with WZB117 inhibited tumors without causing significant adverse events in treated animals.

**Table 1:** Molecules targeting metabolic pathways

Molecule	Target	Disease	Clinical (C), Preclinical (P)	Ref
Metformin	ETC, others	T2D	C	[34]
		T1D	C	[53]
		Cancer	C	[54]
		Lupus	P	[39••]
		EAE	P	[40•]
		Allograft rejection	P	[42•]
Bz-423	F1F0-ATPase	GVHD	P	[18]
2DG	Hexokinase	Cancer	C	[55]
		T1D	P	[41••]
		Lupus	P	[39••]
		Allograft rejection	P	[42 •]
WZB117	GLUT1	Cancer	P	[33]
STF31	GLUT1	Cancer	P	[32]
DON	Glutaminase	Cancer	C	[56]
		Allograft rejection	P	[42•]
BPTES	Glutaminase	Cancer	C	[57]
CB839	Glutaminase	Cancer	C	[37]

*2DG* 2-deoxy-D-glucose, *BPTES* Bis-2-(5-phenylacetamido-1,3,4-thiadiazol-2-yl) ethyl sulfide, *DON* 6-diazo-5-oxo-l-norleucine, *EAE* experimental autoimmune encephalomyelitis, *ETC* electron transport chain, *GLUT1* glucose transporter 1, *GVHD* graft versus host disease, *T2D* type 2 diabetes

Mitochondrial metabolism seems to be important for chronically activated T cells. Targeting of mitochondrial metabolism can be achieved by inhibiting the mitochondrial F1F0-ATPase with the benzodiazepine Bz-423 [18]. In a mouse model of bone marrow transplantation and graft vs host disease (GVHD), it was shown that while expanding bone marrow cell relies on glycolysis, T cells proliferating in response to allo-antigens during GVHD increased both aerobic glycolysis and OXPHOS. In the model, the use of the F1F0-ATPase inhibitor Bz-423 induced selective apoptosis in T cells arresting established GVDH without affecting hematopoietic and immune reconstitution. The mechanism by which apoptosis is triggered in activated alloreactive T cells was the increase of superoxide. Even though these findings challenged the current view that activated T cells are dependent from aerobic glycolysis, they also indicate that manipulation of the redox status and mitochondrial activity can be target pathways in novel therapeutic options for immune disorders. Metformin belongs to the biguanide class of drugs and is widely used for the treatment of type 2 diabetes (T2D) [34]. Metformin affects metabolism as inhibitor of the mitochondrial respiratory chain (complex I) and by activating the AMP-activated protein kinase (AMPK). AMPK is an energetic cell sensor that monitors the AMP/ATP ratio and induces the metabolic reaction to energetic stress. This includes the inhibition of mTOR through the phosphorylation of the tumor suppressor TSC2, which inactivates Rheb and dampens mTOR activity. In addition, AMPK also phosphorylates Raptor, which binds to and regulate the activity of mTOR. AMPK dampens anabolic metabolism and increases FAO.

Glutaminase, the enzyme that converts glutamine into glutamate, is the target of several drugs developed to inhibit the process of glutamine oxidation in the TCA. Several drugs acting on glutaminolysis have been developed in cancer research and showed inhibition of tumor growth in vitro and in vivo [35]. Some of these drugs can find an application also in the control of the T-cell response. The glutamine analog 6-diazo-5-oxo-l-norleucine (DON) inhibits glutaminase, but also a range of glutamine-dependent enzymes (such as glutamine fructose-6-phosphate amidotransferase), as well as other glutamine-dependent reactions, making its biological activity nonspecific. Bis-2-(5-phenylacetamido-1,3,4-thiadiazol-2-yl)ethyl sulfide (BPTES) is an allosteric inhibitor of glutaminase that showed minimal off-target effects [36]. Other GLS inhibitors have been developed, including the BPTES-like drug candidate CB-839 and compound 968, which are currently being tested in clinical trials to determine efficacy, toxicity, and side effects in humans [37]. At the moment, CB-839 and compound 968 are the most advanced and promising drugs available to specifically inhibit glutamine metabolism. An alternative option to inhibit glutamine metabolism with a well-known drug would be rapamycin, which is a widely used immunosuppressive drug. mTORC1 activation has been correlated with increased nutrient uptake and metabolism, but no connection to glutaminolysis has been initially reported. However, a recent paper showed that mTORC1 promotes glutamine anaplerosis by activating glutamate dehydrogenase (GDH) [38]. This regulation requires transcriptional repression of SIRT4, the mitochondrial-localized sirtuin that inhibits GDH. Considering the role of mTORC1 activation in inducing glucose metabolism, rapamycin can be considered to be a multi-target metabolic inhibitor, in addition to a wide range of immunological effects. Metabolic inhibitors have been tested in animal models of autoimmunity and transplantation. Systemic lupus erythematosus (SLE) is supported by chronic activation of CD4+ T cells supported by both glycolysis and mitochondrial metabolism. In vivo treatment of B6.Sle1.Sle2.Sle3 (TC) mice with a combination of metformin and 2-deoxy-D-glucose (2DG) led to normalization of T-cell metabolism and reversed disease biomarkers [39••]. Moreover, T cells isolated from patients with SLE also exhibited increased glycolysis and mitochondrial metabolism, and in vitro treatment with metformin slightly reduced IFN-γ production. A study conducted in a mouse model of experimental autoimmune encephalomyelitis (EAE) showed that treatment of mice with metformin reduced pathogenic Th17 cell while increasing the percentage of Foxp3+ regulatory T cells secreting TGF-β and IL-10 [40•]. Metformin treatment attenuates clinical signs of EAE and the mechanism of action involved the suppression of the mTOR pathway and its downstream target HIF-1α. Immune therapy with metabolic blockade has been tested in the non-obese diabetic (NOD) mouse model [41••]. The authors studied diabetogenic CD8+ T cells that recognize a peptide from the diabetes antigen IGRP (NRP-V7-reactive) in pre-diabetic NOD mice and found that they have features of memory T cells, including an increased use of glycolysis and reduced use of oxidative phosphorylation. Blocking glucose metabolism with 2-DG resulted in a reduced frequency of these cells, a reduced islet infiltrate, and in improved β-cell granularity. Metabolic inhibition can be an effective strategy to prevent allograft rejection. In a model of skin or heart transplantation from Balbc into C57BL6 mice, a triple inhibition of glycolysis with 2DG, mitochondrial metabolism with metformin and glutaminolysis with the glutaminase inhibitor 6-diazo-5-oxo-L-norleucine (DON) was proven to be effective in preventing allograft rejection [42•]. Although these findings are still preliminary, metabolic manipulation is becoming a promising therapeutic venue for immune disorders, including autoimmunity.

## Targeting T-Cell Metabolism in Type 1 Diabetes

**Figure 2:**
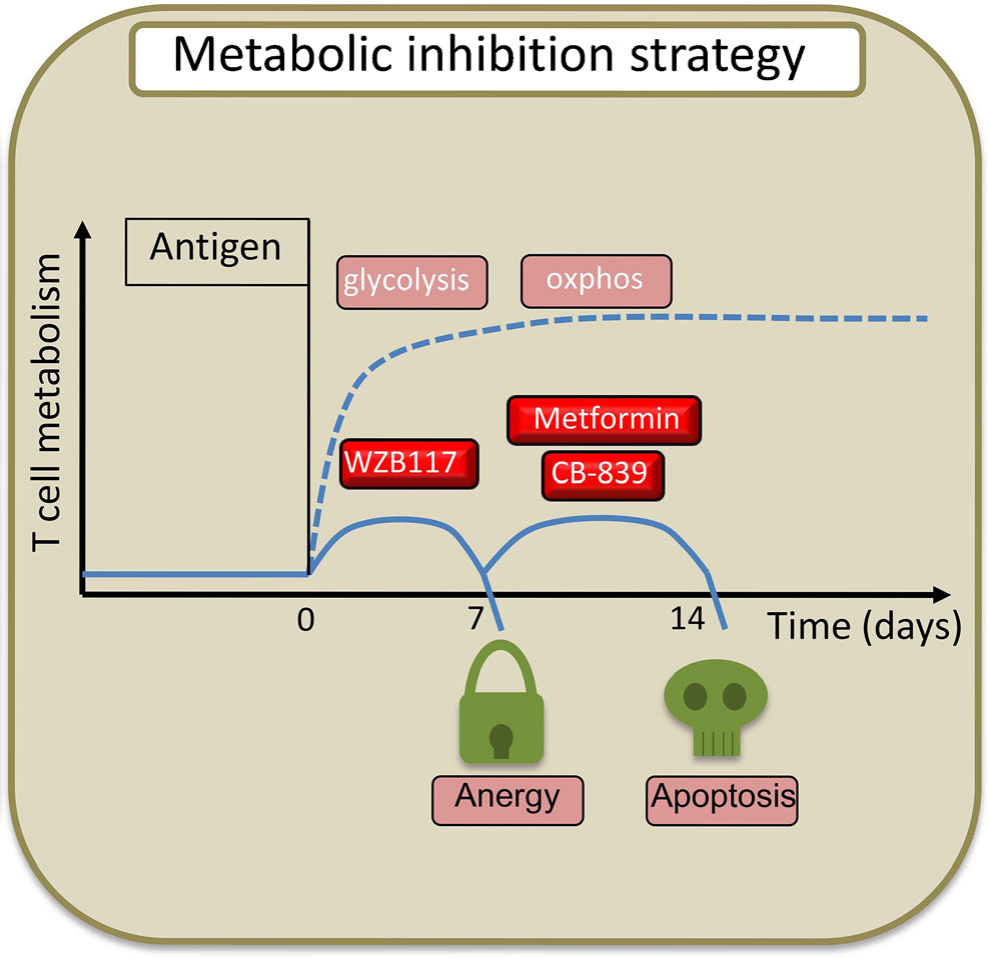
A model for T-cell metabolic blockade. The strategy for metabolic blockade after T-cell activation consists of the inhibition of the glycolytic pathway early after activation and later of the mitochondrial metabolism for 2 weeks in order to induce apoptosis and unresponsiveness selectively in antigen activated T cells. *oxphos* oxidative phosphorylation

With the best of our current knowledge and the availability of novel classes of metabolic inhibitors, how would we design a metabolic inhibition immunotherapy for T1D (Fig. 2)? Our primary goal is to permanently and selectively unarm autoreactive T cells with a short-term treatment. The expectation is that metabolic blockade during antigen specific T-cell activation may induce anergy and apoptosis in activated clones, resulting in a long-term effect on the autoimmune T-cell repertoire. T cells are particularly susceptible to metabolic inhibition right after activation and during the clonal expansion phase. Unfortunately, in the natural history of the disease it is not possible to determine a time window in which quiescent autoreactive T cells get activated by antigens [43–45]. An ideal model is islet transplantation in patients with T1D. In the model, quiescent autoreactive T cells (including memory clones) are re-exposed to β-cell antigens (islet graft) [46] and metabolic inhibition could be envisaged as an induction therapy in this clinical setting. With respect to the natural history of T1D, however, one possibility would be to associate metabolic blockade to antigen specific vaccination with β-cell associated antigens [47] such as GAD65 [48]. The duration of the treatment is an important issue. While metabolic reprogramming is rapid and occurs within hours from antigen recognition, the time sequence for the initial glycolytic burst and the later activation of oxidative phosphorylation last for several days. A metabolic blockade of 2 weeks should be able to cover all the metabolic events of acute and chronic T-cell activation. With respect to the metabolic inhibitors to be used, we envisage a treatment for blocking all the three main pathways: glycolysis, OXPHOS, and glutaminolysis. With respect to glycolysis inhibitors, the best candidate is the specific GLUT1 inhibitor WZB117. Inhibition of GLUT1 has the important advantage of targeting cells that preferentially use GLUT1 for glucose uptake. This includes activated T cells and neurons [49]. Neurons also express GLUT3 that mediates glucose uptake in conditions of low extracellular glucose such as in the cerebrospinal fluid [50]. The redundancy of glucose transporters in neurons should be able to prevent or reduce the possibility of neurotoxic effect using WZB117. When used in the animal model, no neurotoxicity was reported for WZB117 treatment [33]. To inhibit mitochondrial metabolism, the best candidate at the moment is metformin. Metformin is a widely used drug in the treatment of T2D, with a good toxicity and safety profile, and is an efficient inhibitor of the electron transport chain. However, metformin has a wide range of metabolic effects, making it more difficult to predict potential interactions or side effects when used in combination with other metabolic inhibitors. To block glutaminolysis, the best candidate at the moment is the glutaminase inhibitor CB839. Even though the results of the use of CB839 in clinical trials have not been reported yet, inhibition of glutaminase with DON and BPTES showed important side effects and toxicity [51]. Therefore, we expect the use of CB839 to be the most problematic in a metabolic inhibition strategy. An important aspect to be considered in the metabolic inhibition approach is the potential synergies of metabolic inhibitors. Metabolic pathways are highly redundant and the availability of oxygen, and nutrients can rapidly induce a shift in cell metabolism. The simultaneous blocking of the three main metabolic pathways can potentially induce anergy and apoptosis in activated T cells at concentrations far lower than that of the single drugs. With the current knowledge and the therapeutic arsenal of metabolic inhibitors available, we think that a triple metabolic inhibition strategy with WZB117, metformin, and CB839, administered for a 2-week period after antigen-specific activation of autoreactive T cells, is the strategy that conjugates a selective and durable effect on the autoreactive T-cell pool, limiting potential toxicity and side effects. The discovery of novel metabolic inhibitors and their testing in clinical trials could result in important improvements of this strategy in the near future [52].

## Conclusions

Metabolic inhibition as a strategy to control the T-cell response is gaining increasing interest in most of the diseases involving T cells, including transplantation, cancer, and autoimmunity. Immunotherapy for T1D is currently limited by the relative toxicity and side effects of metabolic inhibitors. However, this is a rapidly expanding field of research in pharmacology, and novel drugs may soon be available to design a treatment with a better safety profile. We believe that we are now in the position to start considering this approach, improve our knowledge about the metabolic changes during activation of autoreactive T cells, and set up preclinical models to determine if this strategy is effective for a future translation in clinical immunotherapy.
